# A case report of gastroduodenal intussusception caused by gastric gastrointestinal stromal tumor

**DOI:** 10.3389/fonc.2026.1800737

**Published:** 2026-05-18

**Authors:** Congpu Wen, Shengwei Ji, Maowei Pei, Lili Jin

**Affiliations:** 1 Hangzhou Emergency Medical Center, Hangzhou, China; 2 Department of General Surgery, Qingtian People’s Hospital, Lishui, China; 3 Department of General Surgery, Zhejiang Hospital, Hangzhou, China

**Keywords:** case report, gastric resection, gastric tumor, gastroduodenal intussusception, GIST, laparoscopy

## Abstract

**Introduction:**

Intussusception is most commonly seen in children and is relatively rare among adults. While it can occur anywhere in the gastrointestinal tract, gastroduodenal intussusception resulting from gastrointestinal stromal tumors (GIST) is a rare clinical finding.

**Case presentation:**

Here we report a case of gastroduodenal intussusception caused by gastric GIST in a 68-year-old female. She sought medical attention for symptoms including nausea, recurrent vomiting of blood, and epigastric pain. Plain computed tomography (CT) scan revealed a well-defined mass located on the gastric fundus, which was confirmed intraoperatively. Endoscopy demonstrated that the mass had prolapsed into the duodenum. Based on these findings, gastroduodenal intussusception secondary to a gastric tumor was diagnosed. The patient underwent successful partial gastric resection, which confirmed a mass originating from the gastric fundus and prolapsing into the duodenum. The postoperative pathological diagnosis was low-risk gastric GIST according to the National Institutes of Health (NIH) criteria. The patient was followed up for 12 months, undergoing CT imaging at 6 months and both CT and gastroscopy at 12 months, with no evidence of tumor recurrence.

**Conclusion:**

Gastric GISTs are a rare but clinically significant cause of adult gastroduodenal intussusception, typically necessitating surgical intervention. Multimodal imaging, particularly CT, plays a crucial role in preoperative diagnosis, while histopathological examination remains essential for definitive diagnosis and risk stratification. Treatment should be individualized based on tumor size, location, and patient factors.

## Introduction

Gastroduodenal intussusception is an extremely rare event in adults. The etiologies primarily include gastric tumors such as gastrointestinal stromal tumors (GISTs), carcinoma, lipoma, schwannoma, and leiomyoma according to literatures (1). A comprehensive literature review by Hsieh et al. (2) covering the past two decades identified gastric GIST as the predominant cause, accounting for over half of reported adult cases. Here, we report a case of gastroduodenal intussusception caused by endophytic gastric GIST in a 68-year-old female. We discuss the clinical manifestations, diagnosis, management, and prognosis in the current case within the context of existing literature. A summary of previously reported cases is provided in **Table 1** to contextualize our findings.

**Table 1:** Summary the clinical characteristics of gastroduodenal intussusception caused by GIST in PubMed.

Case no.	Authors	Date of publishing	Country	Age (year)	Gender	Clinical symptoms	Tumor location	Tumor size (cm)	Imaging test	Treatment
1	Siam et al. (3)	2008	Malaysia	29	M	Abdominal pain and anaemia, nausea and vomiting,	Antrum	6×6	EGD	Laparotomy; Partial gastrectomy(Bilroth’s I)
2	Chan et al (4)	2009	China	34	F	Epigastric pain	Fundus	6.5×4.4	CT; EGD	Laparoscopic; wedge resection
3	Gyedu et al. (5)	2011	Ghana	59	F	Intermittent vomiting	Anterior wall	7×6	CT; US	Laparotomy; wedge resection
4	Costi et al. (6)	2011	France	81	M	Abdominal pain and anemia	Posterior wall	4×3	CT; EGD	laparoscopic; Atypical gastrectomy
5	Seok et al. (7)	2012	Korea	51	M	Nausea, vomiting, melena and severe anemia	Antrum	5.5×4.2×1.7	EGD; CT	Laparotomy; Partial gastrectomy
6	Wilson et al. (8)	2012	Ireland	78	F	Epigastric discomfort, vomiting and anorexia	Distal body and antrumc	4.4×3.3×3.4	CT; EGD	Endoscopic and laparoscopic; wedge resection
7	Rittenhouse et al. (9)	2013	USA	52	F	Epigastric pain, vomiting	Fundus	5×5	CT; EGD; US	Laparoscopic; wedge resection
8	Anania et al (10)	2013	Italy	63	F	Epigastric discomfort, dyspepsia and bilious vomiting	Fundus	19×11×9	CT; EGD; US	Laparoscopic; wedge resection
9	Babannavar MSPB et al (11)	2015	India	74	M	Vomiting	Posterior wall		EGD; CECT	Laparotomy; wedge resection
10	Yildiz et al (12)	2016	Turkey	85	F	Epigastric discomfort, nausea, vomiting and weight loss	Fundus	6×5	CECT; US	Laparotomy,subtotal gastrectomy and Roux and Y anastomosis
11	Komatsubara et al. (13)	2016	Japan	90	F	vomiting	Body	5×4.5×4	EGD; CT	Laparotomy, wedge resection
12	Jameel et al (14)	2017	India	65	F	Epigastric pain, vomiting	Posterior wall	6×6×4	EGD; CT	Laparotomy, wedge resection
13	Yamauchi et al (15)	2017	Japan	95	F	vomiting and melena	Posterior wall	4.2×3.9	CT; EUS	Endoscopic; ESD
14	Zhou et al (16)	2018	China	69	M	Abdominal pain, nausea and vomiting	Antrum	4.5×4	EGD; CT	Laparoscopic; wedge resection
15	Ssentongo et al. (17)	2018	Ghana	85	F	Epigastric pain dyspepsia	Fundus	2.5×2.5	CT	Laparotomy, wedge resection
16	De et al. (18)	2018	India	42	F	Epigastric pain and vomiting	Anterior wall	8×7×4	EGD; CT	Laparotomy, wedge resection
17	Đokić et al. (19)	2019	Slovenia	62	M	Vomiting, weight loss	Body	7.5×5.5×4	CECT; US	Laparotomy, wedge resection
18	Mohammed AA et al. (20)	2019	Iraq	65	F	Vomiting, weight loss	Body	45×21	EGD and biopsy; CT	Laparotomy, Total gastrectomy, roux-en-y esophago-jejunostomy and jejunojejunostomy
19	Michael et al. (21)	2021	Uganda	23	F	Epigastric pain, vomiting	Body	6×7	CECT; EGD	Laparotomy; wedge resection
20	Arora E et al. (22)	2021	India	36	M	Anemia, and melena	Body	9.5×8.5×7.5	CT; EGD	Laparoscopic; wedge resection
21	Hsieh YL et al (2)	2021	China	84	F	Postprandial fullness with nausea and vomiting	Body	5.9	EGD; CT	Endoscopic; ESD
22	Numpraphrut P et al. (23)	2022	Thailand	55	M	Epigastric pain and vomiting	Fundus	5.5×4.3×4	CT; EGD; EU	Laparoscopic; wedge resection
23	Zhang W et al (1)	2022	China	85	M	Black stools; epigastric discomfort	Body and antrum	5×3.5	EGD; CT	laparoscopic exploration; Laparotomy; wedge resection
24	Nicola S et al (24)	2022	Italy	36	M	Melena and severe anaemia	Fundus	11×4	EGD; CT	Laparotomy; wedge resection
25	Singh K et al (25)	2023	USA	72		abdominal pain, nausea and vomiting	Body	5.6×5.3	EGD and biopsy; CT	Endoscopic(Failure), Laparotomy, distal antrectomy (Billroth II)
26	Tagliaferri A R et al (26)	2023	USA	85	M	abdominal pain	Body	5.6×5.3	EGD; CT	Laparotomy; Distal gastrectomy (Billroth II)
27	Mujaheed Suleman et al (27)	2024	Tanzania	75	F	abdominal pain, weight loss	Fundus	6x6	CT	Laparotomy, wedge resection
28	Our case	2024	China	68	F	Vomiting blood	Fundus	5×4×3	EGD; CT	Laparotomy; wedge resection

## Case description

A 68-year-old female was admitted to the Gastroenterology Department with a 1-day history of nausea, recurrent hematemesis, and epigastric pain. She had comorbidities of hypertension (amlodipine 5mg daily), cerebral infarction (aspirin 100mg daily), diabetes (metformin 0.5g thrice daily), and intermittent celecoxib use for gallstone-related pain.

Physical examination showed signs of anemia, and a soft abdomen without guarding, rebound tenderness, or palpable masses. Notably, the patient had right limb muscle strength grade 0 (left grade V-). This profound weakness was a long-standing sequela of a prior cerebral infarction that had resulted in persistent right-sided hemiplegia. No other focal neurological deficits were present, and this finding did not directly impact the gastrointestinal presentation but was relevant for perioperative mobility and rehabilitation planning.

Laboratory tests revealed severe anemia (hemoglobin 52 g/L, hematocrit 0.157), leukocytosis (9.99×10^9^/L), and normal CEA, Ca 19–9.

**Figure 1:**
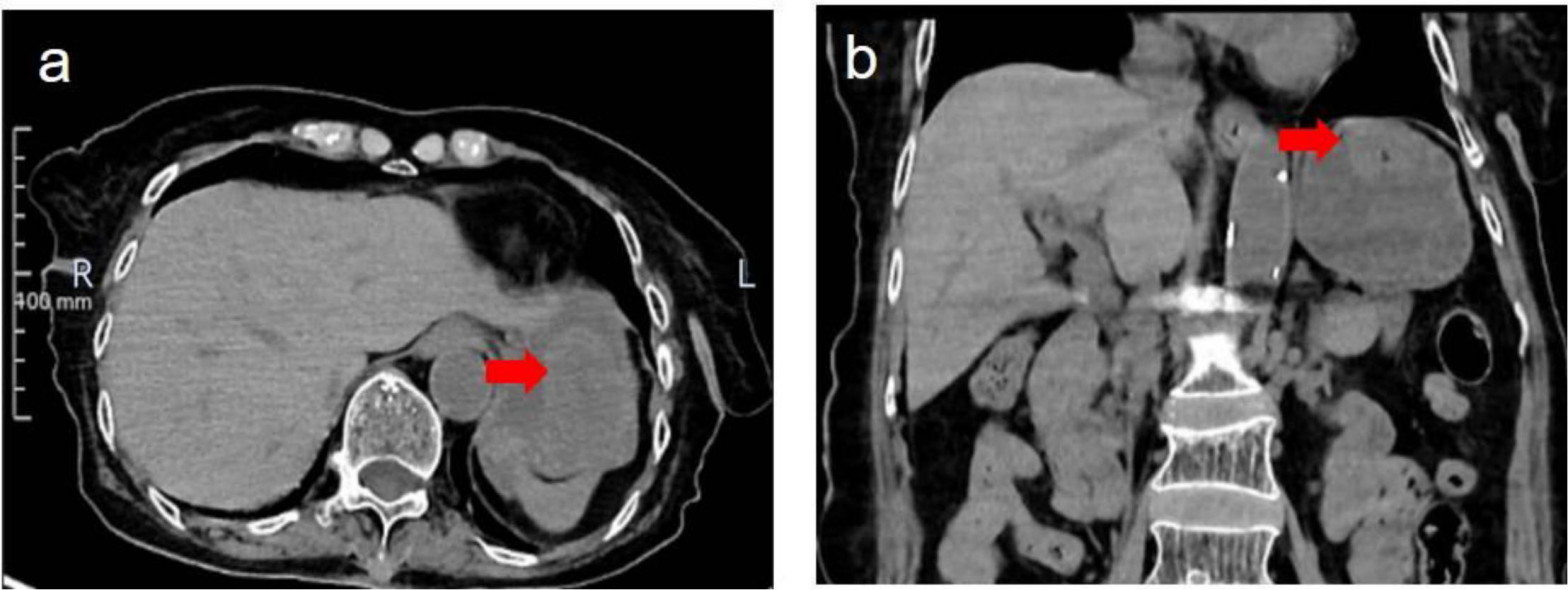
Intraluminal tumor in the gastric funds on CT. Axial **(a)** and coronal **(b)** CT images reveal an intraluminal gastric fundus tumor with well-defined borders (arrow).

Abdominal computed tomography (CT) scan demonstrated a well-defined, intraluminal, heterogeneous mass measuring 5.4×4.8×4.2 cm located at the gastric fundus (**Figure 1**). The mass exhibited soft-tissue density with focal hyperdense areas suggestive of intratumoral hemorrhage, correlating with the patient’s history of hematemesis. No definite signs of intussusception (such as the “target sign” or bowel wall thickening) were seen on CT, but the mass abutted the pylorus. There was no evidence of lymphadenopathy, ascites, or distant metastases.

**Figure 2:**
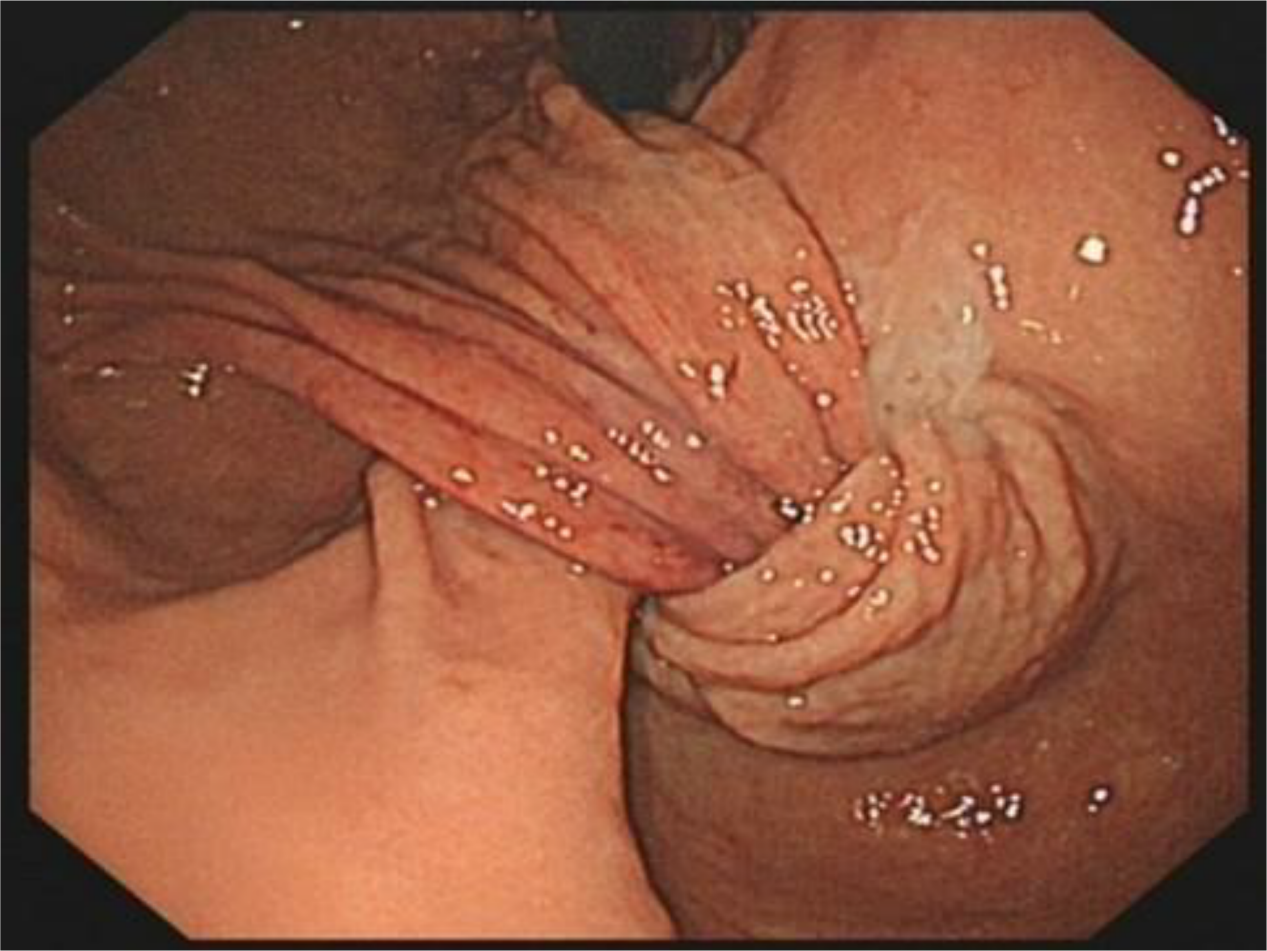
Endoscopic appearance of the gastric mass. Gastroscopy shows mucosal uplifting of the gastric fundus; the tumor caused invagination of the gastric cardia through the antrum and pylorus into the duodenum, resulting in gastric outlet obstruction.

Gastroscopy confirmed a gastric fundus mucosal uplift, with the tumor prolapsing through the pylorus into the duodenal bulb, causing gastroduodenal intussusception and gastric outlet obstruction. Endoscopic reduction was attempted but was unsuccessful (**Figure 2**). Endoscopic ultrasound (EUS) was not performed because the patient presented with active hematemesis and hemodynamic instability, requiring urgent surgical consultation. Moreover, the diagnosis of intussusception secondary to a gastric tumor was already strongly suggested by the combination of CT and gastroscopy, and EUS would not have altered the urgent need for surgical resection.

The patient was transferred for surgery. Diagnostic laparoscopy was converted to laparotomy, revealing a pylorus-impacted gastric mass without abdominal dissemination. R0 radical resection with wide margins was performed after intussusception reduction, and the gastric wall defect was transversely sutured in two layers (no tumor rupture).

**Figure 3:**
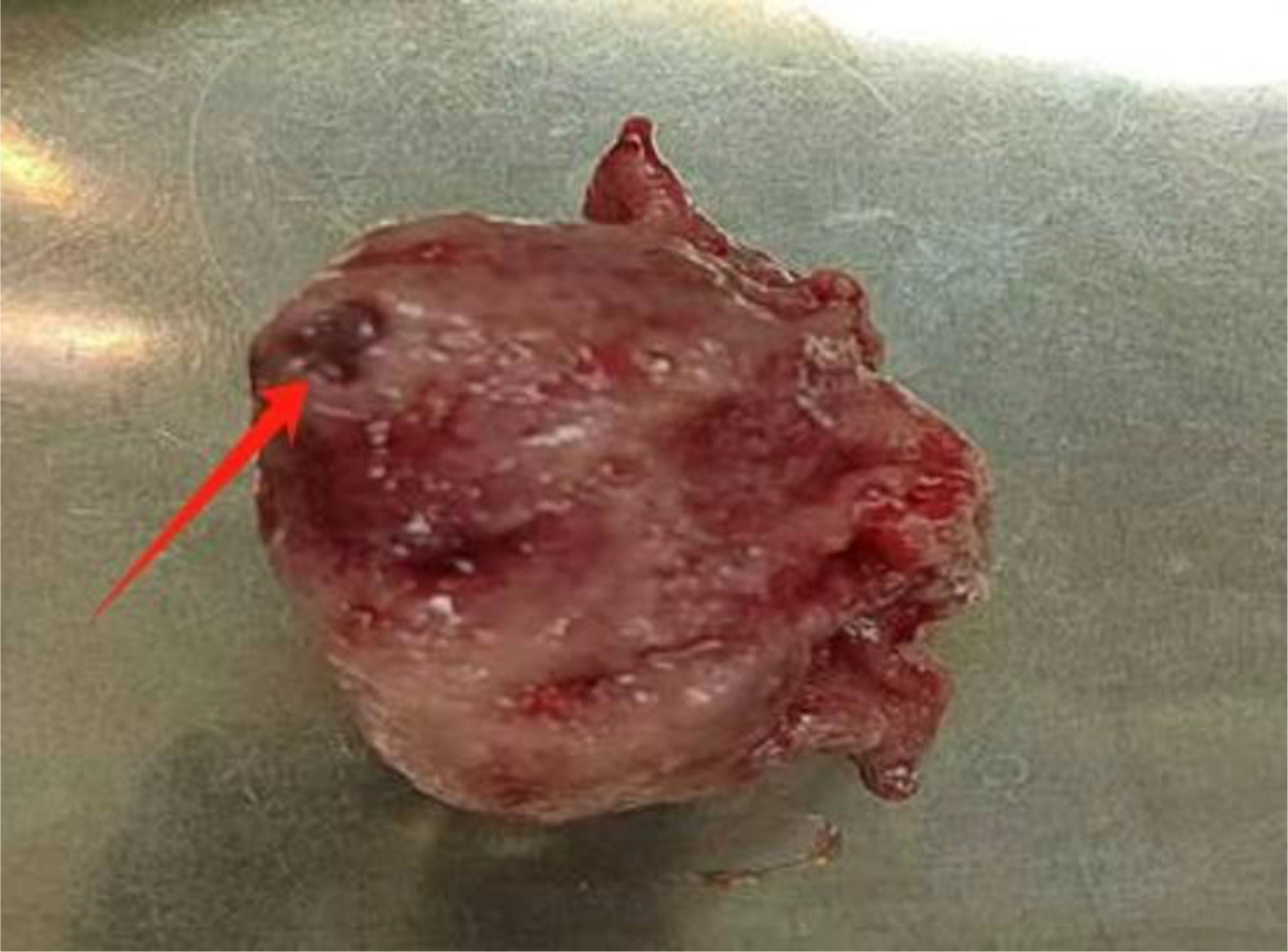
Macroscopic specimen of the excised tumor. The macroscopic resection specimen measures 5 cm × 4 cm × 3 cm and displays hemorrhage spots, cystic changes, and necrotic areas.

**Figure 4:**
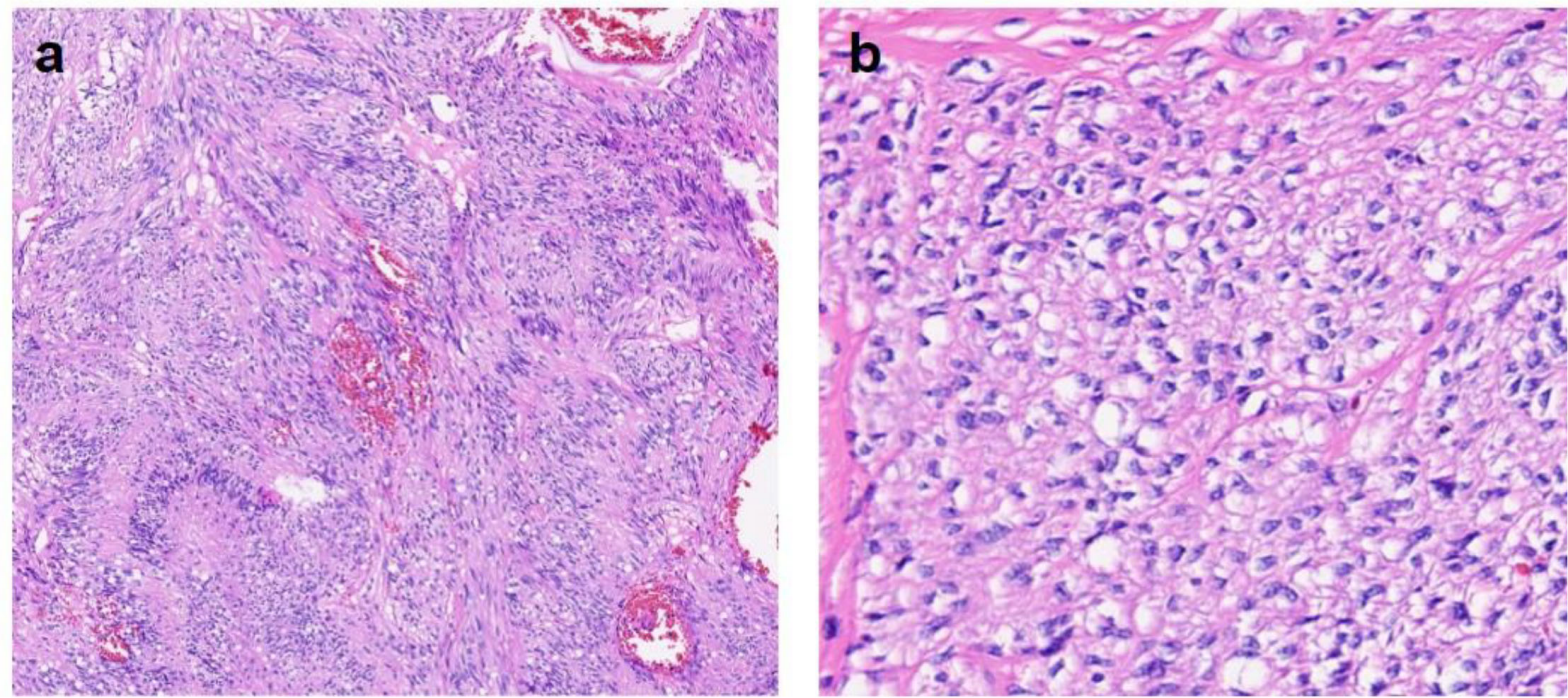
Histological features. **(a)** HE staining (×100) shows a tumor with a visible capsule, hemorrhage area, fasciculate cellular arrangement, and myxoid changes. **(b)** HE staining (×400) reveals fusiform to epithelioid tumor cells with hyperchromatic nuclei, uneven chromatin distribution, karyorrhexis, and mitotic figures. HE, Hematoxylin–Eosin.

**Figure 5:**
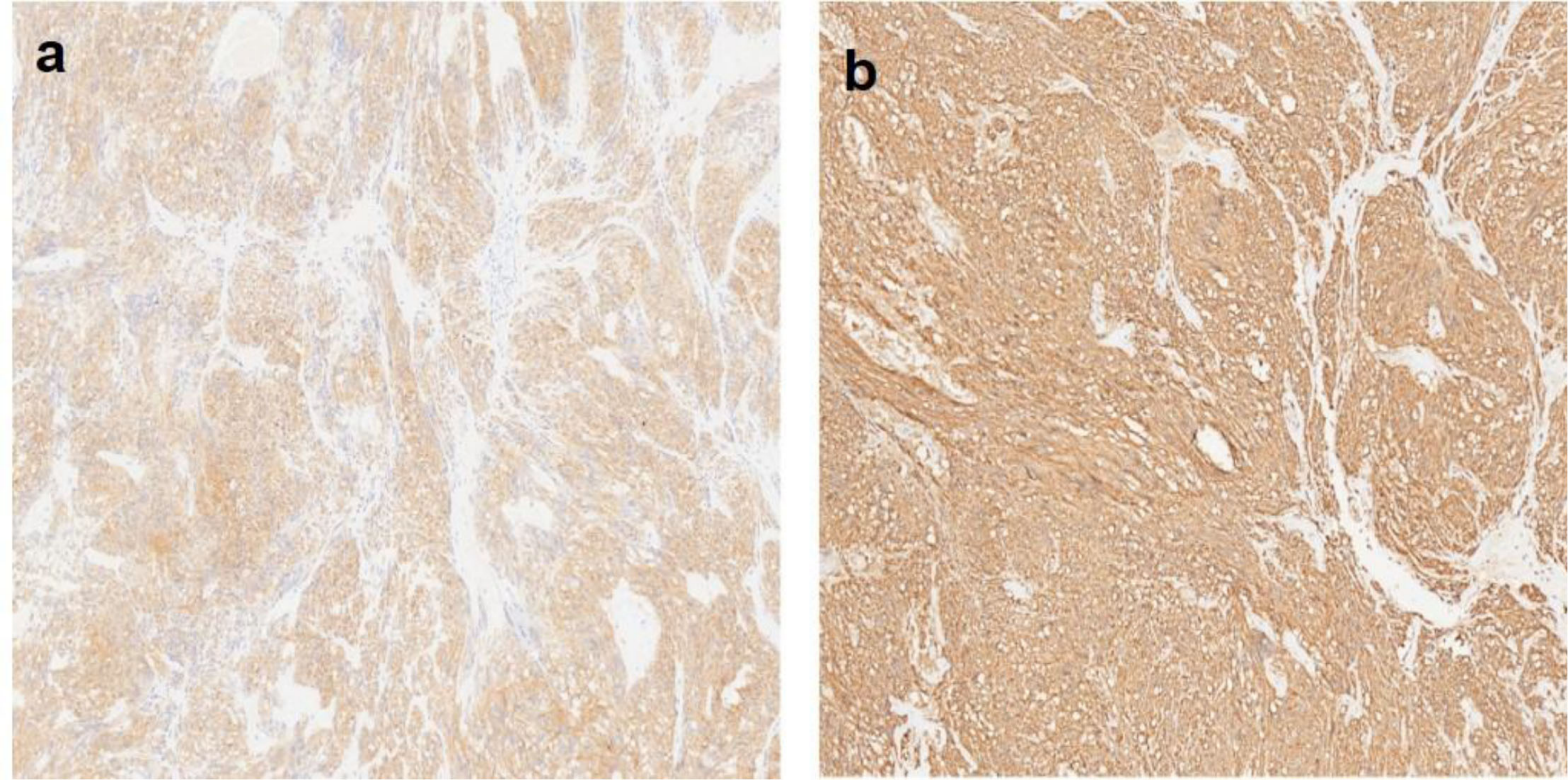
Immunohistochemical features. **(a)** IHC for CD117 (×100) demonstrates strong positivity with clear cell changes. **(b)** IHC for CD34 (×100) shows intense staining in epithelioid tumor cells. CD, cluster of differentiation; IHC, immunohistochemistry.

Postoperative histopathology showed a 5×4×3 cm epithelioid tumor (**Figure 3**) with 2 mitoses/50 high-power fields (HPFs) (**Figure 4**). Immunohistochemistry (IHC) was positive for CD34, DOG-1, CD117, Vimentin, Ki-67 (≈3%), and P53 (**Figure 5**). According to the National Institutes of Health (NIH) consensus criteria for GIST risk stratification (based on tumor size, mitotic count, and anatomical site), the tumor was classified as low−risk (size 5 cm, mitoses 2/50 HPFs, gastric origin). The pathological diagnosis was low-risk epithelioid gastric GIST with intratumoral bleeding. The final clinical diagnosis was gastroduodenal intussusception secondary to gastric GIST.

The patient recovered well and was discharged on postoperative day 8. She was followed up for 12 months, undergoing contrast-enhanced CT at 6 months and both contrast-enhanced CT and gastroscopy at 12 months; no evidence of tumor recurrence or metastasis was observed.

## Discussion

This report presents a case of adult gastroduodenal intussusception caused by a gastric GIST. The clinical validity and methodological accuracy are well-supported by the clear correlation between preoperative findings, surgical observations, and definitive pathology. Preoperative CT and endoscopic findings of an intraluminal mass causing intussusception were directly confirmed during laparotomy. Histopathological analysis confirmed a low-risk epithelioid GIST. The diagnosis was solidified by characteristic immunohistochemistry (CD117+, DOG-1+, CD34+) and low proliferative activity (mitotic count 2/50 HPF, Ki-67 ~3%), fulfilling standard diagnostic criteria. The tumor was classified as low−risk according to the NIH criteria. The consistency between clinical manifestations (gastrointestinal bleeding, anemia, obstruction), imaging, surgical findings, and histopathology ensures the accuracy and internal validity of the case.

The principal unique aspect of this case is the etiology of the intussusception. Adult gastroduodenal intussusception is an extreme rarity, and a GIST acting as the lead point is a seldom-reported cause, with fewer than 30 documented instances in the medical literature (5). Clinically, it diverged from the typical indolent course of many GISTs by presenting as an acute surgical emergency with upper gastrointestinal bleeding and obstruction. A notable feature was the intermittent nature of the symptoms, suggesting episodes of spontaneous reduction and recurrence of the intussusception—a dynamic process rarely elaborated in previous reports. Furthermore, the patient’s multiple comorbidities added significant complexity to perioperative management.

Two interlinked hypotheses can explain the observed clinical and anatomical findings. First, GISTs originate from the interstitial cells of Cajal, the gut’s pacemaker cells. As this epithelioid GIST grew to a substantial size (exceeding 5 cm), it formed a well-defined, intraluminal lead point. Normal gastric peristalsis likely exerted traction on this mass, progressively telescoping the gastric wall through the pylorus into the duodenum. This is supported by the intraoperative finding of the tumor protruding into the duodenum and the classic “target sign” on imaging. The tumor’s low-risk biology (low mitotic rate, well-defined borders) allowed it to achieve this size without prior metastasis or rupture. Second, the “intermittent reducibility hypothesis” accounts for the patient’s fluctuating symptoms (intermittent epigastric pain, recurrent vomiting). Unlike irreversible intussusception seen in children, adult intussusception secondary to GISTs may be transient. Variations in peristaltic force and gut wall elasticity could allow for spontaneous reduction, leading to temporary symptom relief, followed by recurrence. This cyclical pattern explains the patient’s episodic pain and vomiting and is consistent with the absence of peritoneal signs or clear ischemia on exam. This intermittent nature can significantly delay diagnosis, as symptoms mimic more common, transient gastrointestinal disorders. The patient’s severe anemia likely resulted from acute bleeding from ulcerated and necrotic tumor surface mucosa. This ulceration occurs when tumor growth outpaces its blood supply. The tumor’s vascular nature, evidenced by strong CD34 positivity, may have exacerbated the bleeding once the mucosal barrier was breached.

This case offers several critical lessons for diagnosing and managing rare gastrointestinal presentations. In terms of diagnosis, this case emphasizes the necessity of considering GIST-induced intussusception in adults with upper gastrointestinal symptoms, particularly episodic pain or vomiting accompanied by anemia or bleeding. Given the non-specific clinical manifestations, clinicians should avoid misdiagnosing such cases as common gastrointestinal disorders. CT (6)should be the first-line investigation due to its ability to visualize the characteristic signs of intussusception (e.g., target sign, sausage-shaped mass) and to evaluate tumor size, location, and anatomical relationships. In the present case, although plain CT did not definitively show the intussusception, it clearly identified the gastric fundus mass with features of intratumoral hemorrhage, guiding further endoscopic evaluation. While endoscopy directly visualizes the intraluminal mass and can identify surface ulceration, it may miss submucosal lesions. Endoscopic ultrasound (EUS) is highly valuable in such scenarios, providing detailed anatomical layering and enabling fine-needle aspiration (FNA) (7) for a definitive tissue diagnosis before surgery. However, in acutely unstable patients with active bleeding, as in our case, EUS may be deferred in favor of prompt surgical intervention. This multimodal approach can reduce diagnostic delays, which are common in rare diseases and may lead to adverse outcomes.

In terms of management, surgical resection remains the gold standard for GIST-induced intussusception, particularly when endoscopic reduction fails. The surgical goal is complete resection with wide negative margins (R0 resection) to minimize recurrence risk, while avoiding tumor capsule rupture. For low-risk GISTs, a limited resection without routine lymphadenectomy is adequate. The choice of approach (1)—open, laparoscopic, or robotic—should be tailored based on tumor size, location, and surgical expertise. This case also highlights the imperative of tailored perioperative care for patients with significant comorbidities, requiring a coordinated multidisciplinary team to manage coexisting conditions like hypertension, diabetes, and prior stroke.

In terms of follow-up and prognosis, the case confirms that low-risk epithelioid GISTs have a favorable prognosis with no recurrence or metastasis observed within 12 months of follow-up. Our follow-up protocol included contrast-enhanced CT at 6 months and combined contrast-enhanced CT and gastroscopy at 12 months. This supports current guideline-based follow-up protocols, typically involving periodic imaging (e.g., CT every 6–12 months) for several years. Accurate histopathological and immunohistochemical (8) analysis is paramount for risk stratification, based on size, mitotic rate, and tumor location, remains crucial for guiding therapy, including the use of adjuvant imatinib for higher-risk tumors. For these rare cases of intussusception, long-term follow-up data remain scarce, emphasizing the need for continued reporting to better understand long-term outcomes.

In summary, this case highlights the value of case reporting for rare diseases. It reinforces key clinical principles: maintaining a high index of suspicion for unusual etiologies of common symptoms, employing a systematic multimodal diagnostic approach, adhering to oncologically sound surgical principles, and tailoring management to individual patient comorbidities. A summary of previously published cases (**Table 1**) further strengthens the evidence base. Future research should focus on conducting a systematic review and meta-analysis of all reported cases to identify common clinical features, optimal diagnostic modalities, and best surgical practices for this rare condition.

## Data Availability

The raw data supporting the conclusions of this article will be made available by the authors, without undue reservation.
